# Beta-Barrel Nanopores as Diagnostic Sensors: An Engineering Perspective

**DOI:** 10.3390/bios14070345

**Published:** 2024-07-16

**Authors:** Rani Wiswedel, Anh Thi Ngoc Bui, Jinhyung Kim, Mi-Kyung Lee

**Affiliations:** 1 Critical Diseases Diagnostics Convergence Research Center, Korea Research Institute of Bioscience and Biotechnology (KRIBB), Daejeon 34141, Republic of Korea; raniwiswedel@kribb.re.kr (R.W.); anhbui@kribb.re.kr (A.T.N.B.); oz7306@kribb.re.kr (J.K.); 2 Department of Proteome Structural Biology, KRIBB School of Bioscience, University of Science and Technology, Daejeon 34113, Republic of Korea

**Keywords:** beta-barrel, nanopore, diagnostics, protein engineering, biomarker

## Abstract

Biological nanopores are ultrasensitive and highly attractive platforms for disease diagnostics, including the sequencing of viral and microbial genes and the detection of biomarkers and pathogens. To utilize biological nanopores as diagnostic sensors, they have been engineered through various methods resulting in the accurate and highly sensitive detection of biomarkers and disease-related biomolecules. Among diverse biological nanopores, the β-barrel-containing nanopores have advantages in nanopore engineering because of their robust structure, making them well-suited for modifications. In this review, we highlight the engineering approaches for β-barrel-containing nanopores used in single-molecule sensing for applications in early diagnosis and prognosis. In the highlighted studies, β-barrel nanopores can be modified by genetic mutation to change the structure; alter charge distributions; or add enzymes, aptamers, and protein probes to enhance sensitivity and accuracy. Furthermore, this review discusses challenges and future perspectives for advancing nanopore-based diagnostic sensors.

## 1. Introduction

Despite significant advances in the past three decades, the fundamental challenge of accurate and prompt disease diagnosis remains. In particular, the diagnostic fields of cancer [1], emerging viral diseases [2], and mixed microbial infections [3] are in need of faster, more sensitive testing methods that can easily be implemented in low-resource settings. Portable nanopore devices offer a potential solution and can be applied in various clinical settings [4,5,6]. Originally based on the Coulter Counter principle, nanopore sensing provides single-molecule-based ultra-sensitivity compared to traditional diagnostic methods, such as the enzyme-linked immune sorbent assay (ELISA) and polymerase chain reaction (PCR)-based tests.

**Figure 1:**
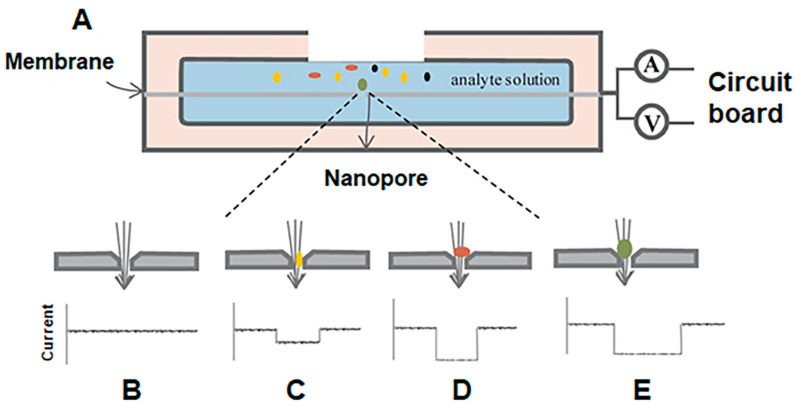
Basic scheme of nanopore-based sensing. (**A**) Two chambers are filled with an electrolyte solution and a voltage is applied across the system. Analytes, represented by multi-colored circles, are then added into one chamber and as each analyte passes through the aperture in the direction shown by the arrow, both dwell times and intensity of current blockades change based on the size and shape of the analyte (**B**–**E**). Reproduced with permission [7].

Nanopore sensors consist of two chambers filled with electrolyte solution, which are separated by an insulating membrane containing a single aperture. An electrical potential is applied across the system, causing any particles present in the solution to begin flowing through the aperture. When particles (or analytes) that are bigger than the ions in the solution move across the aperture, the current across the system changes and the nature of that change depends on the size and shape of the translocating particle (Figure 1) [7]. There are two general classes of nanopore sensors, based on the kind of aperture used; “solid-state” pores, which are artificially created by drilling an aperture into a synthetic insulating membrane [8], and biological pores, which are naturally occurring protein porins purified from host organisms.

Solid-state nanopores have been fabricated from a variety of materials, including silicon nitride (SiNx), silicon oxide (SiO_2_), and graphene. In order to create the pore, “opening” and “tuning” methods are used; “opening” describes the process of creating the initial aperture that will form the nanopore, whereas “tuning” adjusts the aperture size and shape as needed. Recent advances in materials science and surface chemistry have allowed the development of more robust and highly sensitive solid-state nanopore sensors [7,9,10]. Solid-state nanopores are stable, can easily be integrated into devices, and their length and diameter can be precisely controlled, making them ideal for large-scale production as diagnostic sensors [8]. Recently, solid-state nanopores have been utilized for the detection and discrimination of various pathogens. For instance, they have been used to detect the SARS-CoV-2 virus via the incorporation of DNA aptamers [11] and to measure individual hepatitis B virus capsids [12]. On the other hand, biological nanopores offer the significant technical advantages of reduced signal noise and high-resolution analyte detection and have already been commercialized for nanopore-based long-read sequencing by Oxford Nanopore Technologies plc. The MinION portable nanopore sequencing device has been tested for use in the diagnosis of Ebola [6], meningitis [5], salmonella [13] as well as chikungunya, the hepatitis C virus [14], and the SARS-CoV-2 virus [15], demonstrating its practicality in the field.

Furthermore, biological nanopores have been explored as potential diagnostic sensors due to their well-characterized 3D structure and superior capacity for engineering [16,17,18,19,20,21,22,23]. The α-hemolysin pore (α-HL), for example, has been engineered for optimized sensing since 1996, when its sequencing properties were first discovered [24]. This mushroom-shaped pore’s narrow constriction is particularly well-suited to the study of single-stranded DNA or polypeptides, and so has been utilized for DNA sequencing [25], analysis of polypeptide-pore interactions [26], the sensing of organic molecules [27], and more [28,29]. Since α-HL, many new biological pores have been used as nanopore sensors, allowing for a greater variety of analytes to be studied.

Biological nanopores can be categorized into pore-forming toxins (PFTs) and β-barrel pores. PFTs can then be further subdivided into α-PFTs and β-PFTs [30], depending on whether their sensing region consists of α-helices or β-sheets. Although both types of pores are used as nanopore sensors, β-barrel-containing pores (or β-barrel porins) pioneered the field [24], and have a robust, stable structure that is particularly well-suited to modifications [31]. For example, the β-barrel region of α-HL is a popular engineering target to improve the performance of nanopore analysis. Numerous on-pore and off-pore modifications have been reported. Different chemical components, such as adapters [27], aromatic [32], and charged residues [33], can be used to implement on-pore strategies. On the other hand, external biomolecular agents can be employed to detect analytes through various chemical and biomedical interactions like host-guest [33], protein-ligand [34], and enzymatic proteolysis [32]. Some β-barrel porins also have the unique ability to undergo spontaneous gating, caused by the movement of flexible loops or corks that temporarily occlude the entrance of the pore. Although little is known about the precise gating mechanism or how salt concentration, applied voltage, and charge affect it [35], this feature has been and will continue to be a central focus of molecular engineering strategies. β-barrel porins can, therefore, be described as a unique class of nanopores with great potential as highly sensitive, stable, and diversifiable diagnostic tools.

There have been a number of strong reviews published surrounding diagnostic applications of general nanopore sensors including β-barrel nanopores, nanopore-based sequencing, and nanodiagnostics [36,37,38,39,40]. In this review, we focused on engineering strategies for β-barrel porins only, for the purpose of creating diagnostic sensors with enhanced efficacy. We highlight recent progress in protein engineering of β-barrel porins, including the basic structure and charge modifications needed in order to turn a wildtype β-barrel porin into a nanopore, as well as how enzymes, protein “probes,” and DNA “aptamers” may be added to the nanopore in order to create a sensor that can be used to diagnose critical diseases. Thereby, we hope to address four key questions: (1) In what ways can we engineer β-barrels to improve upon their structure and utility as sensors? (2) How do we go about these engineering techniques? (3) Which of these engineering strategies have already been employed to create nanopore sensors to diagnose diseases? Finally, (4) are there any relevant strategies in question 1 that have not yet been employed for the development of diagnostic sensors, and should they be? Finally, future engineering techniques for β-barrel porins will be briefly suggested as a glimpse into the future of this technology and its continued potential.

## 2. Modifying the Structure and Charge of β-Barrel Porins

### 2.1. Modifying Pore Structure

The structure of pores largely determines their sensing capabilities and, therefore, their feasibility for diagnostic applications. There are two main characteristics that should be considered when choosing an appropriate pore for a sensing study; the diameter of the pore’s narrowest constriction (unless a sensing probe is attached to the pore, which will be discussed later) and, for β-barrel porins, the movement of gating elements. Zuo et al. showed the importance of considering nanopore diameter by demonstrating that there is a positive correlation between the size of the target analytes studied and the protein nanopores chosen to study them. This is due to the diameter of the pore’s narrowest constriction, often called the sensing region, as it determines whether a target molecule will firstly be able to translocate the nanopore, and secondly, whether that translocation induces an identifiable signal change. Target molecules should be small enough to enter the pore, but not so small that when they do enter, the change to the ionic flow is negligible [30]. This concept is well explained by Varongchayakul et al.’s elephant-and-ant-through-a-doorway metaphor [22]. Therefore, to analyze target molecules of varying sizes, one must ensure that pores with sensing regions of various sizes are available and that these pores can be engineered so that the diameter of their sensing region is adjustable.

**Figure 2:**
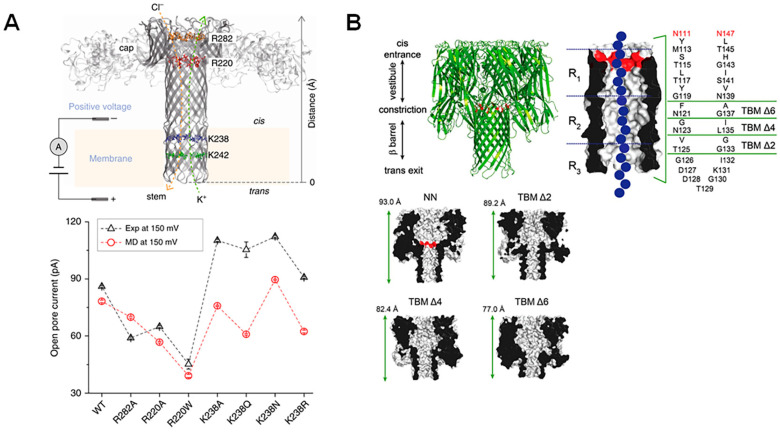
Point mutations and large-scale truncations of nanopores. (**A**) Schematic representation of the aerolysin pore with mutated regions highlighted. Open-pore current at the applied voltage (+150 mV) is plotted for single-channel experiments (black) and molecular dynamics simulations (red), showing the effect of each point mutation. Reproduced with permission [41]. (**B**) Truncated barrel mutants of the α-HL pore used for nucleic acid sensing, which included two amino acid mutations to neutralize charges at the α-HL barrel entrance (shown in red). The regions deleted in order to create the new pore constructs as well as an illustration of a DNA molecule traversing the pore (in blue) are represented in a detailed schematic, while structural representations of the new pore constructs TBM Δ2, Δ4, and Δ6 with adjusted sizes are shown. Reproduced with permission [44].

The introduction of point mutations to nanopore proteins is one possible strategy, as it is relatively simple, but has dramatic effects on structure. For example, the diameter of the aerolysin pore’s narrowest constriction can be controlled from 5 Å to 15 Å by only introducing a single mutation at K238 (Figure 2A), allowing for precise manipulation of analyte dwell time [41]. Similar point mutations have also been explored in α-HL nanopores. In this case, the size and number of sensing zones was reduced by further adjusting the 4S mutant (where three residues in the primary constriction region of the wildtype pore are mutated into serine residues) to incorporate three additional mutations at L135, T125, and D127, all in the β-barrel entrance [42].

However, more dramatic mutations can also be introduced to adjust β-barrel porin structures, as these pores have been shown to be exceptionally stable and so can accommodate these adjustments. For example, several truncated versions of α-HL were found to display similar properties to the wildtype pore [43], but show a sharpened recognition of nucleoside monophosphates (Figure 2B) [44]. Therefore, the introduction of point mutations as well as more significant pore deletion-alterations are feasible and can diversify and improve nanopore sensing capabilities.

A unique feature of the β-barrel porins is the presence of an element on the pore that produces a “gating” effect. In some cases, these elements are attractive features for the conjugation of analyte sensing probes [18,45,46], but in others, they contribute to additional signal noise and are removed [47,48,49,50]. Thus, when considering β-barrel porins for a nanopore study, one must consider whether further engineering of gating elements is necessary. OmpG and FhuA, used mostly for protein sensing, are both well-characterized pores with prominent gating elements [35]. OmpG boasts seven flexible loops on its outer rim, but the movement of its longest loop, L6, is the primary cause of its gating pattern [51].

**Figure 3:**
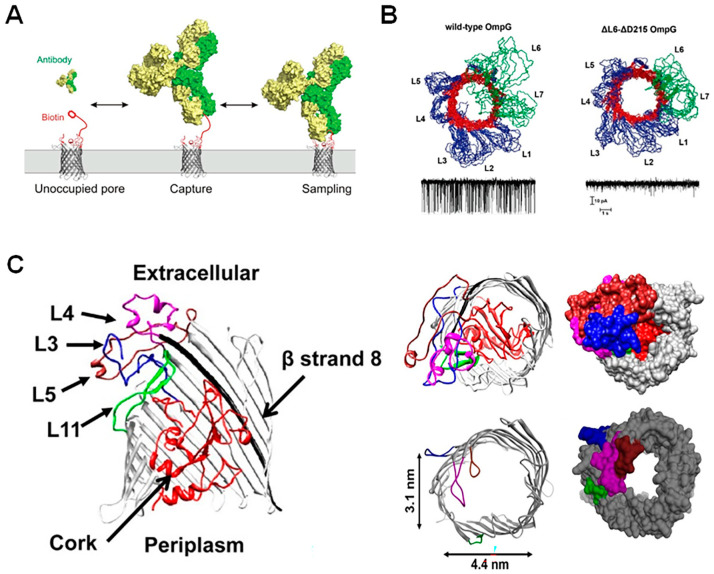
Deletions and restrictions of residues for enhanced nanopore sensing. (**A**) OmpG conjugated to a biotin molecule via a PEG linker tethered to loop 6 allows for the capture and sampling of biotin-binding antibodies in solution. Reproduced with permission [19]. (**B**) Nuclear magnetic resonance ensemble structure of wild-type and loop 6 partial deletion OmpG mutants with resultant wild-type and “quiet” current trace represented below. In the OmpG structure, β-sheets are shown in red, the loops L6 an L7 are shown in green and loops L1–L5, as well as the N- and C-termini and periplasmic turns are shown in blue. Reproduced with permission [53]. (**C**) Schematic representation of wildtype and mutated FhuA, with regions targeted for mutation shown in color; loop 3 (blue), loop 4 (magenta), loop 5 (brown), loop 11 (green) and the cork domain (red) (**left**). The WT-FhuA pore contains a “cork” region that completely occludes the pore entrance (**top right**) and has, therefore, been removed along with loop regions in order to optimize nanopore sensing (**bottom right**). Reproduced with permission [48].

In the last decade, the loops of OmpG have been used as tethers for sensing molecules, equipping this nanopore with greater sensing capabilities (Figure 3A) [19]. However, removing or restricting L6 to produce a “quiet” pore (Figure 3B) allows for accurate sensing ability in a wider range of conditions [52,53], indicating that there may be as-yet unidentified advantages to loop deletion in this system. For FhuA, on the other hand, removal of its cork and extracellular loops is essential for an analyte to traverse the pore [48]. The most commonly used form of this pore is the tFhuA (or TL-FhuA) mutant, as it demonstrates significantly enhanced unitary conductance. In this mutant, the 160-residue cork domain and five extracellular loops (L3–L5, L10, and L11) were deleted, and a 6-His tag with a [PA]3 linker was added to the N-terminus (Figure 3C). Although it is not clear which mutation specifically allows for the reported high unitary conductance, Thakur et al. postulate that it is most likely due to an accidental increase in the diameter of the pore entrance [54]. Since this discovery, Movileanu et al. have shown that, by attaching specific monobodies to the N-terminus of the protein, the tFhuA pore can be used as a generalized sensor for biomarkers in serum [21,50].

### 2.2. Modification of Pore Surface Charge

**Figure 4:**
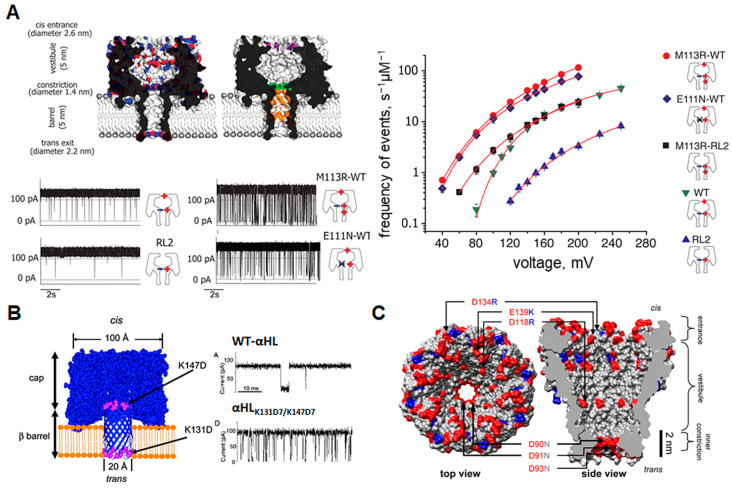
Charge modifications of β-barrel nanopores. (**A**) Models of wild-type (**top left**) and mutated (**top right**) α-HL pores with charge distribution as well as mutated sites shown. Red indicates negative charges, blue indicates positive charges, while mutated sites are shown in purple, green, and orange. Raw current traces after each mutation show how the introduction of positively charged residues into the pore lumen changes the translocation frequency (gating) of DNA (bottom right). The frequency of translocation events at each applied voltage plotted on a logarithmic scale for each pore construct is shown on the right. Reproduced with permission [55]. (**B**) Exit and entry traps (in purple) for polypeptides placed in the α-HL pore and resultant current trance in the presence of Syn B2 polypeptide showing increased translocation of the peptide after pore mutation. Reproduced with permission [58]. (**C**) Structural representation of the MspA pore with mutations introduced to alter charge characteristics for enhanced DNA capture. Blue indicates positively charged regions while red indicates negatively charged regions. Positively charged arginine and lysine were added to the pore vestibule and entrance while three aspartic acid residues were replaced with asparagine to neutralize the inner constriction. Reproduced with permission [62].

The charge characteristics of each pore must also be considered, as electrostatic interactions between analyte and pore play an important role in nanopore-based sensing. The translocation of DNA through wildtype α-HL, for example, is very rare [55]. Therefore, in order to increase the efficiency of the DNA readout, charged groups at the entrance and within the constriction of the pore must be altered. Interestingly, adding positive charges to the constriction region results in significantly increased translocation frequency and lowers the voltage threshold for translocation, despite the fact that free-floating DNA strands would not be affected by a charged group so far inside the pore. A closer examination of the DNA translocation mechanism reveals that DNA strands in the wildtype pore enter and exit the top end of the β-barrel at high frequency, but when positive charges are added to the constriction zone, the strand is pulled through the pore, forcing it to pass through the sensing zone, causing a higher frequency of complete translocation events (Figure 4A) [55]. Further manipulation of the charge environment inside α-HL has since been shown to keep the DNA strand “trapped” within the lumen of the pore for longer, making DNA readout easier [56]. Electrostatic traps have also been applied to controlling peptide translocation (Figure 4B) [26,57,58], which will be of increasing value in diagnostic applications, as peptides become an important indicator for disease states. The MspA pore, an attractive sensor for single-molecule DNA sequencing [59], has undergone similar mutations to those of α-HL. In this case, positively charged amino acids were added to the constriction and vestibule regions in order to increase sequencing capacity (Figure 4C) [60,61]. This increased the total residence time of nucleic acids in the sensing zone [62], much like in α-HL. In recent research, positively charged amino acids in cytotoxin K and aerolysin nanopores were replaced with acidic-aromatic residues in the β-barrel region. This modification allowed effective detection of mixtures of trypsinated peptides at low pH [63]. Similarly, for the purpose of Alzheimer’s disease identification, a modified aerolysin nanopore, in which a neutral threonine was replaced by a positively charged lysine at position 232, enhanced the electrostatic capture and prolonged the residences within the pore of the acetylated and phosphorylated Tau peptides [64]. Thus, decorating the interior surface, particularly the constriction zone, with charged residues has a valuable impact on the residence time of analytes, improving the general sensitivity of the nanopore readout. In summary, when designing a robust nanopore sensor from a β-barrel porin, one must ensure that the size of the constriction region is appropriate for the size of the analyte, that elements that produce gating effects do not occlude binding or translocation signals and that the sensing zone is sufficiently decorated with charges so as to facilitate target molecule interactions.

## 3. Functionalization of Nanopores with Enzymes

Moving on from modifications of the pore itself, we come to the more complex realm of conjugating enzymes to nanopores. Enzyme units can be added to nanopores in order to engineer new functions into the pore, such as the ability to fold, unfold, or proteolytically cleave target molecules [65,66]. Functionalization of nanopores with enzymes is a challenge, as it is difficult to predict whether such chimeras are able to retain the original function of their components. However, by making use of the inherently modular property of proteins, researchers may be able to slot different functional units together like Lego blocks, creating new protein products with enhanced functionality [65].

### 3.1. Choosing an Enzyme

**Figure 5:**
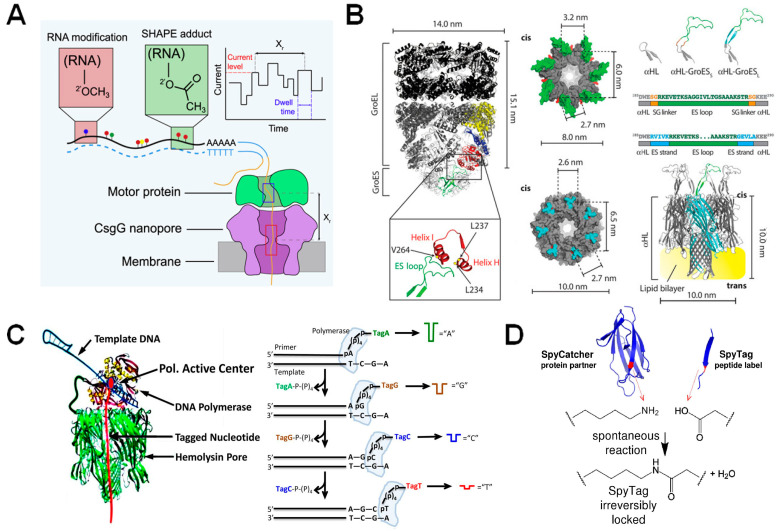
β-barrel nanopores functionalized with enzymes. (**A**) Representation of a motor protein in complex with a beta-barrel pore for the identification of RNA modifications and structure via the SHAPE reagent. Reproduced with permission [68]. (**B**) Nanopore-GroES chimera developed for examining GroEL binding kinetics. Schematic of GroES/GroEL complex with ES loops magnified and labeled (**left**). GroES (**top**) and α-HL (**bottom**) structures shown to the right of the GroES/GroEL complex, with their seven-fold symmetry highlighted. Scheme showing the addition of functional GroES loops onto α-HL and below it, the complete nanopore-GroES pore (**right**). Reproduced with permission [65]. (**C**) DNA polymerase attached to an α-HL pore via the SpyTag/SpyCatcher linker (**left**) and the resultant Sequencing by Synthesis (SBS) workflow (**right**). Reproduced with permission [71]. (**D**) SpyTag/SpyCatcher workflow. The SpyCatcher and SpyTag domains are separated, but upon missing undergo a spontaneous reaction that results in irreversible locking of the two domains in solution. Reproduced with permission [72].

Many nanopore studies are currently focused on protein and DNA sequencing. Consequently, several research groups have added free-floating enzymes into the nanopore chamber or ligated enzymes to target molecules, in order to linearize and control analyte translocation through the nanopore. ClpX, for example, is part of a proteasome complex in *Escherichia coli* and has been used to linearize and translocate proteins through the nanopore at a rate slow enough for ultra-sensitive analysis via its ATP hydrolysis mechanism. By adding ClpX units into the nanopore chamber, the tagged ubiquitin-like protein Smt3 was unfolded and sequenced using controlled translocation through the α-HL pore. For translocation, the enzymatic rate must be carefully controlled; ClpX was chosen for its ability to translocate 80 amino acids per minute across the pore, which is well-suited for nanopore sensing. Additionally, the degree of mechanical force was considered; ClpX generates a force of ~20 pN, which is sufficient for protein denaturation [67]. In another study, a motor protein was ligated to a target RNA strand in order to detect both endogenous and exogenous RNA modifications at specific locations in the strand, as well as investigate RNA structural ensembles in long RNAs. Here, the motor protein was also used to slow down the translocation of the RNA molecule so that modifications could be accurately sampled by the pore. Interestingly, it was found that changes in the translocation rate of the motor protein correlate to sites in the RNA strand where modifications had occurred, thereby providing an additional layer of information (Figure 5A) [68]. Finally, DNA polymerase enzymes have also been used with MspA and α-HL pores to aid in DNA sequencing by controlling the rate of DNA translocation and causing serial replication of target DNA templates [60,69].

For enzymes that facilitate both unwinding and movement of a nucleic acid strand through the nanopore, both structural and functional properties must be optimized. The phi29 DNA polymerase, for example, demonstrates superior ability in nanopore sequencing due to its ability to stably remain atop a nanopore at relatively high voltage and its ability to remain highly processive without the presence of accessory enzymes [70]. These aspects are essential when determining which enzymes can function as accessories to nanopores. Thus, there is clearly a growing need for more functionally complex nanopore sensors to improve sequencing and sensing, and the enzymes used in the above studies are ideal candidates for engineering enzyme-linked pores.

### 3.2. Functionalization of Protein Nanopores with Enzymes

As mentioned previously, an enzyme-linked nanopore can be a complex protein to engineer, but as structural information becomes more readily available and engineering techniques advance, the development of these protein chimeras becomes more feasible. One case of the successful engineering of a biological nanopore with enzyme function is the conjugation of the co-chaperonin unit GroES to α-HL (Figure 5B) [65]. However, Ho et al. state that creating the GroES- α-HL chimera was contingent on both proteins being heptameric and having flat, toroid faces with similar dimensions, thus it is unlikely that this approach can be applied to a broad range of pores and enzymes.

In a more recent case, the membrane-spanning β-barrel of the anthrax protective antigen pore of *Bacillus anthrax* was attached via a hydrophobic SSG linker to proteasome activator 28α (also called REG), creating the REG-nanopore [66]. This new nanopore was then fused with a 20S proteasome from *Thermoplasma acidophilum*. Fusion of these two units was possible by examining the crystal structure of the 20S proteasome in a complex with PA26, a homolog of REG. In this structure, tails extending from PA26 associate with the terminal leucine residue of the 20s proteasome α-subunit. This association was used to complex the same α-subunit with the C-terminal residues of REG that corresponded most closely to the tail residues in PA26. Using this system, a thread-and-read as well as a chop-and-drop approach was developed. In the thread-and-read approach, an additional unfoldase VATΔN was added to the solution, and the protease was deactivated, allowing target proteins to be threaded through the REG-nanopore after initial processing by the unfoldase. In the chop-and-drop approach, the activated protease cleaves an unfolded target protein, while REG-nanopore identifies the resultant peptide segments. The output of this system is a protein fingerprint similar to that produced by mass spectrometry. This study shows that soluble proteins with a toroidal shape may be used as nanopores by conjugation to a β-barrel transmembrane segment via an SSG linker. Furthermore, the endogenous function of fused proteins can be successfully retained during these modifications [66].

In a final example, the α-HL pore was engineered with DNA polymerase function for the purpose of developing a DNA sequencing-by-synthesis system (Figure 5C). In this case, the polymerase was conjugated to a single subunit of the pore, using the SpyTag/SpyCatcher system [71]. The SpyTag/SpyCatcher system requires that two domains (SpyTag and its partner, SpyCatcher) be fused to the binding partners. Upon mixing, the Spy domains rapidly form an irreversible amide bond, locking the two binding partners together with a reaction that shows high tolerance to a range of pH values, buffers, and temperatures (Figure 5D). This system can also be genetically fused to any protein of interest, demonstrating its versatility for a wide range of experiments [72]. Looking back at the above studies, several factors should be considered when engineering chimeric-nanopores as diagnostic sensor machines: (1) structure and symmetry of functional units, (2) protein solubility of the enzyme unit, and (3) methodological convenience in the construction of the enzyme-nanopore chimera.

### 3.3. Diagnostic Applications

It is interesting to imagine the various possibilities for diagnostic sensors with this technology. Thus far, there have been limited diagnostic applications for this technology, and so here we present several future possibilities for the use of this technology in this realm. DNA sequencing forms the basis of diagnosing mixed microbial infections [3] as well as mRNA-based diagnostic tests, and so any addition to pores (such as ATPases, motor proteins, or DNA polymerases) that will increase the speed and accuracy of sequencing will be useful for diagnostic sensing of microbial genomic targets. In the realm of protein sensing, one could examine the possibility of using the unfoldase enzyme as a method for sensing larger biomarkers with relatively small pores. Although the attachment of the unfoldase enzyme was originally intended for sequencing proteins [67], it may be feasible to use an unfoldase to alter the tertiary structure of biomarker proteins in order to decrease the diameter of these targets. This would allow nanopores to detect large proteins as they pass through the pore. Finally, by using the versatile SpyTag system, as described above, one could look at adding other enzyme units to pores, such as exonuclease; by attaching this enzyme over the entrance of a nanopore, one can identify deoxynucleoside monophosphates in the order from which they are cleaved off the chain as a method of DNA or RNA sequencing [73]. Therefore, adding enzymes to nanopores may assist in sequencing nucleic acids faster and more accurately and identifying large protein biomarkers.

## 4. Modification of Nanopores with Aptamers

Aptamer-modified nanopores offer significant advantages in the area of sensing and diagnosis [74], due to their excellent characteristics. Aptamers consist of short oligonucleotide sequences made up of either DNA or RNA that display a strong binding affinity with a particular genomic target. Their small size of 25 to 80 bases is compatible with the nanoscale of biological nanopores and can be chemically modified for simple functionalization on the surface. Furthermore, aptamers exhibit high binding affinity towards a range of targets such as small ions, proteins, and biomarkers, expanding their potential applications, and because of their ability to adopt a flexible 3D shape, aptamers can induce nanopore current changes through triggering binding to target compounds. Finally, the production of these molecules is also cost-effective, making them ideal for pore engineering studies conducted in low-resource settings.

Aptamers are normally screened using SELEX (systematic evolution of ligands by exponential enrichment). From a wide collection of randomly generated oligonucleotide sequences, the specific aptamer is selected and amplified until the strongest binding affinity with the target is achieved [75,76,77].

### 4.1. Choosing an Aptamer

**Figure 6:**
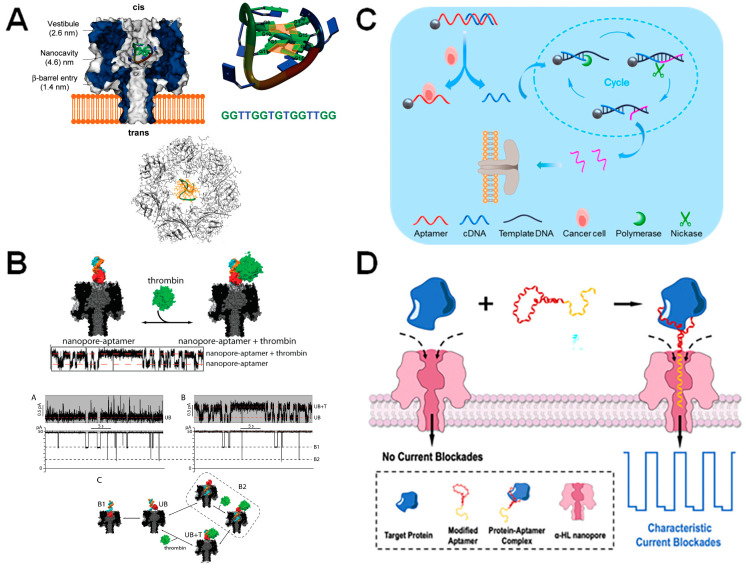
α-HL nanopores equipped with aptamers. (**A**) α-HL pore encapsulates G-quartet TBA (thrombin aptamer). Reprint adapted with permission [82]. (**B**) α-HL nanopore engineered with a thrombin-binding aptamer at its entrance. The thrombin aptamer (red) is hybridized to oligomerA (blue) via a hybridization domain (orange). OligomerA is attached to α-HL via a hexamethylene linker. A raw current trace showing the nanopore signal when thrombin binds and is released from the aptamer is shown below. Reproduced with permission [83]. (**C**) Human lymphoma cancer cell detection using aptamer-coated particles in combination with enzymatic amplification of DNA. The aptamer, which is immobilized onto a bead (shown by the grey sphere) is conjugated to a partially complementary DNA molecule (cDNA). However, when a Ramos cell is bound to the aptamer, the cDNA molecule is released and then undergoes enzymatic cycling amplification. The concentration of output DNA (shown in pink) is then identified via the aerolysin nanopore and correlated to the number of Ramos cells in the solution. Reproduced with permission [89]. (**D**) Aptamer-assisted capture and detection of the SARS-CoV-2 nucleocapsid protein using α-HL. Reproduced with permission [92].

The flexibility of the 3D conformation of aptamers determines the specificity of target recognition. Therefore, it is worth considering the structure of aptamers prior to selecting a candidate for engineering. Aptamers have a negatively charged backbone, causing them to favor interactions with the positively charged surface of target proteins. For example, both thrombin and heparin use their positively charged surfaces to bind to RNA aptamers [78]. Structural analysis also reveals that the nucleic-acid-binding domain of NF-κB and MS2 protein binds to RNA aptamers with a similar affinity to their optimal DNA target [79,80]. In this case, the electrostatic interactions are the most important factor contributing to high binding affinity between aptamers and molecules of interest. Moreover, several studies have been conducted to examine the stability of the aptamer structure using unmodified nanopores to determine the proper aptamer structures for detection. “Y-shaped” aptamers and DNA hairpins were analyzed by measuring the current blockage generated by the α-HL nanopore when interactions occur between aptamers and target analytes or the nanopore itself [81]. The G-quadruplex, which is a folded state of the thrombin-binding aptamer (TBA), was confined within the nanocavity of α-HL for investigating single-molecule folding and unfolding rates [82]. Through measuring the change of α-HL pore conductance, the authors were able to identify folded G-quartet TBA molecules before spontaneous conversion into linear TBA as they travel through the β-barrel under the influence of voltage (Figure 6A). Thus, the aptamer structure and the electrostatic interactions between aptamers and the chosen target should be carefully considered during the design process.

### 4.2. Aptamer-Functionalized Nanopores

The majority of research on aptamer-based engineered nanopores has primarily focused on solid-state nanopores or nanomaterials, due to the capacity of artificial nanopores for manipulation. Nevertheless, there have also been reports of constructing aptamer-functionalized biological nanopores. Rotem and colleagues developed a technique for identifying thrombin by utilizing an aptamer-modified α-HL pore [83]. A 15-mer DNA aptamer that binds to thrombin was connected to the mutated α-HL pore near the entrance with a disulfide bond to a cysteine residue. First, the cysteine mutation was introduced to the wild-type pore at position 17 (α-HL N17C). Attachment of single DNA oligonucleotides to α-HL was performed by mixing the α-HL N17C monomers with an activated thiol-modified single nucleotide (5′ TTTTGCTCACGTTCGCAT 3′, oligoA). Heteroheptameric pores were constructed by mixing N17C monomers with wild-type monomers, followed by the addition of rabbit red blood cell membranes to trigger the assembly of two types of monomers. In order to detect thrombin, oligonucleotides were hybridized to the DNA adapter. The duplex formed between oligoA and aptamer T4 was used to detect thrombin. After adding the thrombin aptamer, two blockade events were observed by analyzing single-channel current recordings. The first level is generated by the movement of the quadruplex domain into the vestibule of the pore, while the second level arises from the insertion of the dsDNA into the pore (Figure 6B). However, the authors propose that the linker between pore entrance and aptamer should be shortened in future research, as it was difficult to distinguish the thrombin binding signal in pores with aptamerT7 (contains a seven-thymine linker) and aptamerΔ3 (contains a 4-nt thymidine linker).

“DNA-nanopores” are a new class of engineered pores used in the detection of single-base mismatches in DNA strands [84]. In this case, α-HL was reconstructed with a single-stranded DNA oligonucleotide covalently attached within the pore lumen. The 5′-thiol-modified oligonucleotide is tethered via a hexamethylene linker and a disulfide bond to α-HL N17C. Single-channel current recording revealed that the binding between DNA molecules carrying a single base substitution and the tethered DNA strand causes changes in the current blockage. In addition, the event lifetime is dependent on the type of mismatch. In practice, this system allows the detection of a drug-resistance-conferring mutation in individual DNA strands of the reverse transcriptase gene of HIV, and the sequencing of a complete codon based on the match/mismatch-dependent binding time of hybridized oligonucleotides. Therefore, Howorka et al. have reported the ability of a single oligonucleotide coupled- α-HL N17C to respond to individual binding events with oligonucleotides of a complementary sequence [85].

### 4.3. Diagnostic Applications

Recently, there has been rising interest in using aptamer-coated nanoparticles with nanopore sensors for diagnostic purposes. The Healey group, for example, has used this method to create a system for rapidly measuring the amount of prion PrPC proteins, which plays a role in neurological disorders [86], while Li et al. altered magnetic nanoparticles by attaching aptamers that specifically bind to varcinoembryonic antigen (CEA), a pancreatic cancer biomarker [87]. In 2020, another study demonstrated the identification of lung cancer biomarkers VEGF, PDGF-BB, and thrombin using three different nanoparticle-coated aptamers and complementary DNA constructs [88]. Lately, Xi et al. also developed a technique for detecting Ramos cancer cells involved in human lymphoma with high sensitivity (Figure 6C) [89]. Using the thrombin-aptamer-decorated ClyA nanopore, authors were able to mimic the nuclear pore complex selectivity for translocated molecules [90]. Following this, detecting protein biomarkers via an external DNA aptamer-probe that shows affinity for specific proteins has attracted great attention [91], with a recent application being the capture of the nucleocapsid protein of SARS-CoV-2, allowing rapid and label-free detection of protein concentrations as low as 10 pM in one hour (Figure 6D) [92].

In summary, aptamers can be utilized as a free probe for detection or attached to the nanopores as an aptamer-functionalized nanopore. Both methods exhibit advantages over conventional biosensors that use aptamers, like aptasensors. Nanopores enable stochastic detection at the single-molecule level, offering additional information about the concentration of target molecules in solution.

## 5. Modification of Nanopores with Protein Probes

**Figure 7:**
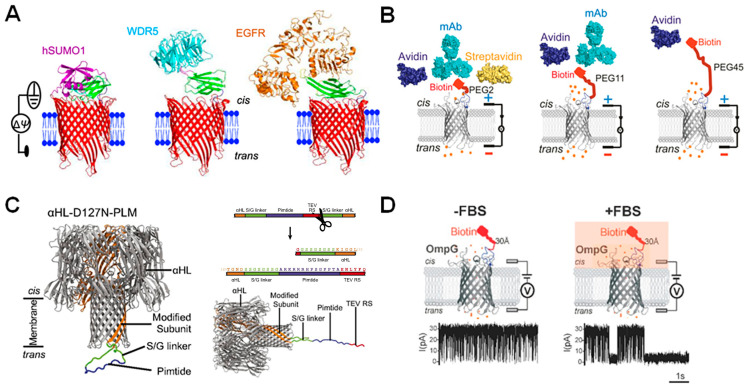
Using β-barrel nanopores equipped with peptide probes for sensing. (**A**) Three modified tFhuA nanopores (red) in a lipid bilayer (blue) containing a monobody (green) that binds to hSUMO1 (purple), WDR5 (blue), and EGFR (orange), respectively. Reproduced with permission [21]. (**B**) OmpG connected to biotin probe via 2, 11, and 45 PEG units showing that increased PEG linker length decreases sensitivity of the nanopore construct for analytes. Structures of biotin binders are shown above. Reproduced with permission [93]. (**C**) Structure of modified α-HL pore with Pimtide bound (**left**) and cleaved (**right**) from the modified subunit and an illustration of the sequence of the cleavable linker added to the modified α-HL subunit, with TEV cleavage site shown in red. Reproduced with permission [99]. (**D**) Schematic representation of OmpG-biotin-linked pores in the absence and presence of fetal bovine serum, with representative current traces shown below. Reproduced with permission [45].

A common strategy for engineering nanopores to detect target molecules is the addition of protein probes to the pore surface. Protein probes are used to attract specific molecules to the nanopore so that they may be identified in a heterogenous solution and their concentration determined, such as in detecting concentration changes of oncogenic proteins in human serum samples [18]. The protein probe in this case must be highly specific for its target to ensure that non-specific interactions between the probe and other serum or buffer components are minimized. The β-barrel porins can also be conjugated to monobodies for diagnostic purposes. The conjugation is mediated through a single cysteine introduced into the nanopore lumen or terminal regions. Recently, monobody-based nanopore sensors were developed for the detection of biomarkers in biofluid. tFhuA nanopores tethered with monobodies derived from the 94-residue fibronectin type-III (FN3) domain were used to make SUMO-tFhuA, Mb4-tFhuA, and Adnectin-tFhuA sensors for detection of hSUMO1, WRD5, and EGFR, respectively (Figure 7A) [21].

Previous work has focused on conjugating protein probes to the OmpG [18,19,45,46,93,94,95], α-HL [96,97], and FhuA [21,50,98] pores, using a variety of methodologies. These include attaching a PEG linker to the pore [18,19,29,45,93], engineering peptides so that they extend directly from the pore lumen as a “tethered bait,” often via a GS-tether [21,50,98,99], or embedding peptide sequences directly into elements of the pore itself (Figure 5A,B) [46,94,97,100].

### 5.1. Choosing a Protein Probe

Probe charge and probe length as well as linker length and charge are important considerations for ensuring that the nanopore sensor can function optimally. The charge of the probe relative to the surface of the pore can influence gating dynamics and thus the total time the pore spends in the open state (τ_on_), while the probe length has been shown to influence drag force through the solution and analyte-pore interactions. Therefore, both aspects can significantly affect sensitivity and selectivity. When conjugating the highly basic Pimtide sequence to the charged *trans* face of α-HL, for example, the point mutation D127N had to be introduced, which neutralized the pore face enough to free the probe from electrostatic entrapment [99]. In the case of PEG-linked pores, linker length determines nanopore sensitivity. Chen et al. found that the further a probe-analyte binding event occurs from the pore, the less likely that event will be translated into an identifiable signal change (Figure 7B) [93]. In line with this, Hwang et. al. report that PEG2-linked pores produced a discernible analyte-binding signal when probing for the apoptosis inhibitor Bcl-xL using the Bak peptide, whereas longer PEG-linkers (such as PEG6 and PEG12) were unsuccessful [18]. However, these effects may be pore-specific so more studies are needed in MspA, α-HL, and FhuA before a general conclusion can be made.

### 5.2. Protein-Probe Based Nanopores

As mentioned above, there are three general methods for adding a protein probe to a nanopore; (1) a PEG-linker, (2) creating a tethered “bait” and (3) embedding the probe within the pore. Understanding the advantages of each method is essential for efficiently engineering nanopores. Using a PEG-linked probe removes the need to genetically modify the pore itself, providing a more high-throughput strategy, as the pore can be purified in bulk, the solution can be separated and then conjugated to many PEG linkers of varying lengths and with different probes for streamlined testing. However, in order to extract PEG-linked pores, the protein solution must be subjected to a chemically-harsh gel separation technique, which may cause proteins to misfold and significantly reduce the overall yield of viable pores. On the other hand, creating bait probes or embedding probes within the pore does not require this process. These two techniques are in essence very similar, with the only distinction being that the bait technique includes a posttranslational cleavage site on one end of the probe, so that this end may be cut in order to allow the bait to extend out from the pore (Figure 7C). When comparing the two techniques, Harrington et al. reported a minimal difference in analyte dissociation rates. However, it was shown that the binding kinetics of the bait probe were more comparable to those of other studies [99], possibly due to embedded probes and their bound analytes being under the electrostatic influence of the nearby pore entryway. Thus, the tethered bait technique for pore sensors likely provides more accurate results. However, the embedded probe technique is useful when considering multiplexed sensors, such as those recently developed in the OmpG nanopore [94], as the physical constraints of the pore structure may allow probes to be more physically separate from each other and the pore lumen, preventing unwanted interactions within the engineered pore.

### 5.3. Diagnostic Applications

Protein probes attached to nanopores have become popular for sensing common cancer-associated biomarkers [18,21,99], as protein expression is typically challenging to detect due to the high signal-to-noise ratio and frequency of non-specific binding. One challenge with the use of probe-conjugated nanopores, however, is that the presence of serum proteins occasionally causes blockage signals that can be confused with true-analyte binding events (Figure 7D) [98] or lead to decreased sensitivity of the nanopore, as long blockages can prevent the identification of binding signals.

**Figure 8:**
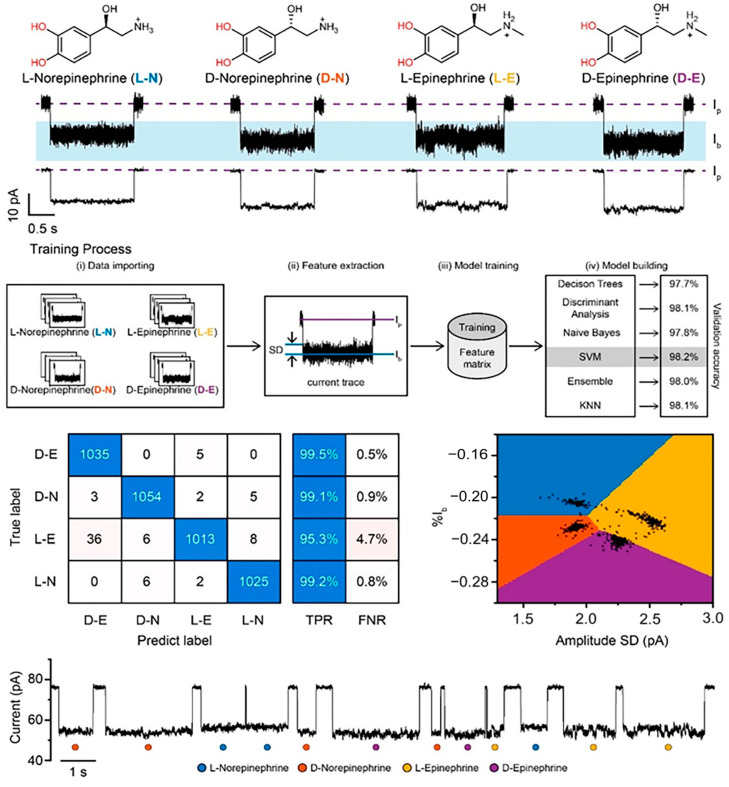
Machine-learning-assisted distinction of nanopore signals from enantiomeric chemicals. Each enantiomer is shown at the top, with the current trace corresponding to their presence shown below. The training process used to tailor the machine-learning algorithm is represented, with the resultant confusion matrix and event classification distinction shown based on the chosen support vector machine, or SVM, model. A representative raw current trace showing events and which enantiomer they represent according to the SVM model is shown at the bottom. Reproduced with permission [102].

However, the rise of machine-learning algorithms may offer a viable solution; where the human eye may not be able to accurately distinguish between specific and non-specific events, unsupervised machine-learning algorithms can provide assistance. Recently, a machine learning workflow was created to successfully distinguish enantiomers using a modified MspA pore with an accuracy score of 98.2%, despite the molecules only differing in handedness (Figure 8). In another example, the detection of α-synuclein-derived peptides bearing different post-translational modifications (PTMs) was enhanced significantly using deep-learning-assisted single-molecule detection with engineered aerolysin nanopores. The deep-learning-enhanced recombinant K238A aerolysin nanopore allowed the detection of α-synuclein PTMs in the clinical samples of patients with various neurodegenerative diseases such as Parkinson’s disease [101]. A similar workflow could be used to identify non-specific serum interactions from the binding signals of target molecules.

Furthermore, nonspecific blockages by serum components have been reported in some studies [45,98], but their presence has not been consistently documented [18,19,46], leaving it unclear to what extent this phenomenon might interfere with the accuracy of nanopore sensing. Should this become a more pronounced problem, it may also be necessary to consider a degree of patient sample pre-processing, such as the removal of large macromolecular complexes or high-concentration components from sera, before nanopore analysis.

In summary, protein probes have great potential as components of diagnostic sensors, with studies already underway to implement these systems for early cancer diagnosis. By ensuring probes do not interact with the electrostatic surface of the pore, linker length is kept relatively short, and appropriate data analysis techniques are used to distinguish true binding signals, protein-probe-conjugated pores have the potential to become part of a new wave of highly sensitive, accurate diagnostic biosensors.

## 6. Conclusions and Further Prospects in Pore Engineering for Diagnostics

β-barrel pores can serve as sturdy nanostructures due to their robust structural integrity and high thermodynamic stability. Additionally, their sensitivity to interactions with other molecules results in accurate readings. These characteristics allow for the creation of effective single-molecule sensors that can be used in a range of applications, including biomedical diagnostics. With the rapid advancement of basic research, an increasing number of β-barrel porins are being redesigned to meet ongoing needs and address technical limitations in nanobiotechnology.

In this review, we briefly summarized how β-barrel porins are redesigned to address persistent demands in the diagnosis and prognosis of numerous human diseases. First, nanopores can be modified in the context of structure and charge distribution to diversify and improve sensing capabilities. Protein mutagenesis such as point mutations and deletions can manipulate the pore diameter, shape, and electroosmotic and electrophoretic force characteristics, as well as the stability of pore insertion into bilayers. Second, incorporating enzymes, DNA aptamers, and protein probes through chemical covalent linkers or fusion strategies offers additional approaches to target a variety of molecules and address the issue of detection limitations.

Despite the many advantages, utilizing β-barrel biological nanopores for sensing remains difficult due to limited pore size and interfering characteristics, such as gating elements that occlude the pore entrance. Additionally, sensitivity and accuracy are the main obstacles when using biological nanopores for detecting biomarkers in clinical samples, due to the large amount of impurities present. To address these drawbacks, numerous engineering techniques can be used to improve existing pores before the discovery of novel pores becomes necessary. Specifically, the utilization of hybrid nanopores, light-controlled nanopores, programmable nanoreactors, and nanopores embedded in a single live cell are alternative approaches to aid the detection of analytes via β-barrel porins. For example, a hybrid nanopore featuring engineered OmpG and a bilayer molybdenum disulfide (MoS_2_) solid-state nanopore was developed to minimize the noise levels of detection for high-resolution biomolecule sensing. Using the hybrid nanopore instead of the solid-state nanopore results in a signal-to-noise ratio improvement of approximately 1.9 times and dwell times that are about 6.5 times longer [103]. In addition, using light to control a pore channel may offer a more fine-tuned sensing technique without compromising biological systems [104]. Programmable nanoreactors for stochastic sensing are currently a cutting-edge technology. This approach bypasses the complications associated with pore engineering by introducing a synthetic strand of chemically engineered nucleic acids to the pore lumen in order to identify enantiomers or chemical intermediates [102,105]. Furthermore, the use of machine learning in nanopore analysis can enhance the precision of complex data interpretation and lower the expenses associated with manual data analysis [106,107,108]. For example, utilizing the electro-osmotic flow trap produced by an asymmetric electrolyte combined with the MspA nanopore and a machine-learning algorithm, it is possible to accurately distinguish lysozyme, apo/holo-myoglobin, and the ACTR/NCBD protein complex, which have varying charges, with 99.9% precision [106].

Artificial intelligence (AI)-assisted signal analysis has also been used to identify sub-components of complex mixtures with significantly higher accuracy than with manual analytical methods. The structurally similar sub-components of gentamicin have been successfully distinguished using a convolutional-neural network model [109], semi-supervised classification has identified specific kinin biomarkers at physiological serum concentration [110] and RNA modifications have been identified using custom machine-learning algorithms [111,112]. Since promising early results using logistic regression and random forest and k-nearest neighbor algorithms [113], machine-learning-assisted nanopore analysis has made a valuable impact, specifically in the separation of analyte signals from noise and in the identification of structurally similar compounds [109,111,114,115,116].

Embedding a nanopore in a single live cell for DNA secretion or neurotransmitter sensing holds promise for advancing drug discovery and neuron sensors in the future [117]. Real-time detection of the L-Glu was successfully demonstrated using the M2MspA-N91H nanopore incorporated in a live cell membrane. This sensor shows steady single-channel behavior and responsiveness to various locations of L-Glu binding.

qPCR, a conventional technique, showed a limit of detection (LOD) of at least ~1 CFU/mL of DNA or ~0.025 ng of DNA, although this may vary depending on the source of the DNA [118,119]. Previous studies of real-time PCR detection or comparing nanopore and real-time PCR reported that LODs of Oxford Nanopore Technology-based amplicon sequencing were one-to-two orders of magnitude lower than real-time PCR (~2.5–50 CFU/mL) [120,121]. The LOD of a nanopore sensor is crucially dependent on the capture rate of analytes. β-barrel biological nanopore engineering such as the introduction of specific probes for diagnostic biomarkers as previously mentioned [18], pore surface charge modification [55] and the optimization of buffer compositions [122] enables increased capture rate of analytes, resulting in enhanced LOD of nanopore sensors.

For efficient use of β-barrel-containing nanopores in diagnostics, we suggest several strategies for protein engineering and data analysis: (1) hybridization of biological nanopores and solid-state nanopores, (2) protein engineering of the transmembrane region to stabilize interactions between pores and membranes, (3) pore surface modification to increase the capture rate of biomarkers, and (4) integration with other techniques such as light-based sensing as well as rapid data analysis using AI-based algorithms. While numerous efforts have been put forth, there is still potential for further advancement of β-barrel-containing nanopore sensors as diagnostic sensors by enhancing factors beyond just stability and accuracy. The reduction of detection speed (sample-to-answer time), discovery of highly specific probes against biomarkers, and development of straightforward and efficient methods for massive nanopore data analysis should be considered. We suggest that single-molecule-based nanopore detection using engineered β-barrel biological nanopores is powerful for the specific detection of biomarkers. With advancements in nanopore-based multi-arrayed systems utilizing engineered protein nanopores, this technology may enable the simultaneous detection of multiple biomarkers in the future, leading to significant improvements in the accuracy and precision of disease diagnostics.
